# Augmented Go/No-Go Task: Mouse Cursor Motion Measures Improve ADHD Symptom Assessment in Healthy College Students

**DOI:** 10.3389/fpsyg.2018.00496

**Published:** 2018-04-11

**Authors:** Anton Leontyev, Stanley Sun, Mary Wolfe, Takashi Yamauchi

**Affiliations:** Department of Psychological and Brain Sciences, Texas A&M University, College Station, TX, United States

**Keywords:** ADHD, executive function, impulsivity, mouse tracking, go/No-go task

## Abstract

Attention deficit/hyperactivity disorder (ADHD) is frequently characterized as a disorder of executive function (EF). However, behavioral tests of EF, such as go/No-go tasks, often fail to grasp the deficiency in EF revealed by questionnaire-based measures. This inability is usually attributed to questionnaires and behavioral tasks assessing different constructs of EFs. We propose an additional explanation for this discrepancy. We hypothesize that this problem stems from the lack of *dynamic* assessment of decision-making (e.g., continuous monitoring of motor behavior such as velocity and acceleration in choice reaching) in classical versions of behavioral tasks. We test this hypothesis by introducing dynamic assessment in the form of mouse motion in a go/No-go task. Our results indicate that, among healthy college students, self-report measures of ADHD symptoms become strongly associated with performance in behavioral tasks when continuous assessment (e.g., acceleration in the mouse-cursor motion) is introduced.

## Introduction

Impulsivity—deficiency in behavioral inhibition (Barkley, 1997; Alderson et al., 2012)—is a key characteristic of attention deficit/hyperactivity disorder (ADHD). It affects many facets of decision-making processes in young children (Geurts et al., 2006) as well as adults (Mäntylä et al., 2012). Valid and standardized measurements of impulsivity are crucial for assessment of ADHD, leading to a long and fruitful discussion about merits and shortfalls of different measurements of impulsivity, and more broadly, executive functioning (EF) in general. Two most commonly used methods are self-report rating scales, which require participants to answer a number of questions about their own life, and performance-based tests, which require participants to complete a certain cognitive task, e.g., respond when presented a particular stimulus.

However, despite being aimed at assessing the same construct, rating scales and performance-based tests show little to no correlation between each other. For example, only a miniscule correlation was observed between performance-based tests’ results and rating scales (Toplak et al., 2013) or occupational functioning measurements such as the frequency of conflicts at work or job terminations (Barkley and Murphy, 2010). In a meta-analysis study, Toplak et al. (2013) show that among reported (182 out of 306) correlations between performance-based measures of EFs and scores on Behavior Rating Inventory of EF (BRIEF) only 19% were significant, with a median correlation value of 0.18. Five (19%) of reported correlations between performance-based measures of disinhibition were significantly correlated with rating-based measures of disinhibition with a median correlation of 0.25. For the Behavioral Assessment of the Dysexecutive Syndrome-Dysexecutive Questionnaire (BADS-DEX), five studies reported all possible relevant correlations (76 correlations in total). Among them only 28 (37%) of these measures were significantly correlated with performance-based measures (a median correlation of 0.14).

In what follows, we first discuss the strengths and weaknesses of different EF measurement tools and, in particular, tools measuring impulsivity. We then lay out different rationales for these weaknesses and propose continuous motor assessment as a potential remedy. Finally, we introduce an experiment that examines the degree to which these augmentations contribute to amending the discrepancy problem.

Rating scales are widely used in diagnostics of EF-related psychiatric and neurodevelopmental disorders such as ADHD, Tourette syndrome, bipolar disorder, depression, obsessive-compulsive disorders, autism, and traumatic brain injury. Longitudinal studies have shown rating scales to be a good indicator of these disorders across one’s lifespan, for they have strong correlations with other measures regarding retention of these disorders. They have also been found useful to discern residual (sub-clinical, when only a handful of symptoms remain) and full-clinical types of disorders when used together with other diagnostic tools such as structured clinical interview. In addition, rating scales were useful in highlighting comorbid disorders, including the comorbidity between ADHD and substance use, anxiety, or antisocial disorders (Biederman et al., 1995).

However, as a diagnostic tool, rating scales have a number of important shortcomings. First, a large portion of shared variance can arise between different rating scales (e.g., questionnaire-based ADHD and EF measures) simply because these rating scales were developed concurrently and administered in a similar manner, providing a potential source of bias (Richardson et al., 2009). As Kamradt et al. (2014) point out, if we rely solely on rating scales for an understanding of ADHD and EF dysfunction, it may make the relationship between EF deficits and ADHD appear stronger and more significant than it really is. Second, there exists a large body of research showing a discrepancy between performance-based tasks and self-report rating scales (Barkley and Murphy, 2010, 2011; Toplak et al., 2013; Solanto, 2014), but not much between performance based-tasks and informant-based rating scales. For example, a study by Burgess et al. (1998) found no correlation between performance-based tasks and self-report rating scale scores in the majority of their cases. However, they found many significant correlations between performance-based task results and informant-based ratings. For instance, participants’ performance for the trail-making test, cognitive samples test, and Modified Wisconsin Card sorting test were all significantly correlated with Dysexecutive (DEX) Questionnaire scores when the scores were obtained from caregivers, but the correlations disappeared completely when the self-report version of the questionnaire was applied. In other words, there is a difference in how individuals perceive their own EF and how their peers perceive it. This discrepancy is particularly worthy of attention in light of studies showing informant-based questionnaires to be a more reliable measure of EF-related disorders, such as ADHD, than self-reports (Barkley and Murphy, 2010).

Performance-based measures have also drawn considerable attention in recent years as an initial tool to gauge individual differences in EF and their aberrations (Barkley, 1997, 2014). Many studies have reported the performance in performance-based tests to be a good indication of disorders of a different nature, from neurocognitive disorders to personality disorders. For example, performance-based tests were found to be indicative of borderline personality disorder (Posner et al., 2002), schizophrenia (Dickinson et al., 2007), and anxiety (Miu et al., 2008).

However, performance-based EF measurements have limited ecological validity (Burgess et al., 2006; Chaytor et al., 2006). The association between self-reported impulsivity ratings such as Conner’s adult ADHD questionnaire (CAARS) or Barkley’s Deficits in Executive Functioning Scale (BDEFS) and go/No-go task performance is often miniscule or even non-existent. An aforementioned meta-analysis of 20 EF studies by Toplak and colleagues showed only 19% of performance-based disinhibition measures to be weakly correlated with rating-scales based measures of disinhibition. Similarly, when compared to the occupational measurements (e.g., number of times a person was fired from a job or quit due to boredom), performance-based measures fared poorly too. Only the Continuous Performance Test and the Kaufman Hand Motion task were found to have a significant relationship with occupational functioning (Barkley and Fischer, 2011).

Why are performance-based measures so poorly correlated with both occupational functioning measures and rating scales? Some researchers suggest that rating scales and performance-based tests assess different concepts of EFs (Anderson et al., 2010; Toplak et al., 2013). The difference between these constructs is best explained from the concept of different cognitive levels (Stanovich, 2011). This theory states that the mind operates on two levels: algorithmic and reflective, as stated by Marr (1982). The algorithmic level is mostly concerned with efficiency of cognitive abilities, such as perception, attention, and working memory. The reflective level, on the other hand, is concerned with long-term features, such as beliefs and goals of the system in general. Only at this level does optimal decision-making come into question. Performance-based measures can only uncover one’s ability to perform in a highly structured, laboratory environment. In such an environment, only optimal performance becomes evident. In contrast, rating scales measure typical behavior, in which individuals decide what to consider optimal by themselves. Some examples of these concepts are “impulsive disinhibition,” measured by performance-based tasks, and “impulsive decision-making,” measured by rating scales and non-performance based tasks, such as delay discounting (Reynolds et al., 2008). It should be noted that the discrepancy between performance-based measures and questionnaire-based measures of ADHD symptoms can be attributed to the change of the symptoms across the lifespan (Lasser et al., 2012).

In addition to these explanations, we think that there are other fundamental reasons for the discrepancy in EF measurement between rating scales and performance-based tasks. First, performance-based EF tests rely primarily on two sources of data: accuracy and response time. Response time shows only the time passed between the start of a trial (i.e., stimulus presentation) and response (i.e., button press), omitting any interactions that happened between these two points. Accuracy measures, due to their binary nature (only “correct” or “incorrect” responses are recorded), are prone to the same shortcoming. As a result, if impulsivity or other type of executive dysfunction impacts performance continuously, any information about these interactions is lost. As further evidence of the importance of the dynamic processes, recent studies have shown that behavioral disinhibition and EF deficiency are best assessed by variability of response times, rather than average response time (Hauser et al., 2016).

Moreover, there is a pitfall in the way data from performance-based tasks is analyzed. Stepwise regression and Pearson’s correlation are the most often used methods; these analyses assume linear relationships between dependent and independent variables, i.e., response time and rating scale scores. However, scores on rating scales and scores of performance-based testing might not be statistically compatible because the distribution and variability of these measurements’ units are likely different. For example, although scores in rating scales are distributed normally, response time measures are known to be highly skewed, making an ex-Gaussian most commonly used distribution for response time (Ratcliff, 1978). This incompatibility between EF tests and rating scales can explain the difference in correlation strength between both ADHD and EF rating scales, and EF rating scales and occupational functioning measures. Taken together, these observations suggest that performance-based tests can be improved by amending response measures.

Remedies can be found in the measurement technique that has recently attracted considerable attention in the field of decision-making: mouse cursor movement analysis. Mouse movement features, such as the area under the curve or the deviation from the straight line between the start and end point, were found to be indicative of perceptual and numerical judgment (Song and Nakayama, 2008; Chapman et al., 2010; Xiao and Yamauchi, 2014, Yamauchi and Xiao, 2017), semantic categorization (Dale et al., 2007), linguistic judgment (Spivey et al., 2005; Farmer et al., 2007), and racial and gender judgment of morphed face pictures (Freeman and Ambady, 2009; Freeman et al., 2010). Additionally, mouse movement has been found to be related to attitudinal ambivalence toward certain topics (e.g., abortion) (Wojnowicz et al., 2009; Schneider et al., 2015), uncertainty in economic choices (Calluso et al., 2015) as well as general emotional states, such as anxiety (Yamauchi, 2013; Yamauchi and Xiao, 2017).

In the context of the current study, one of the most important findings is the relationship between mouse motion and dynamics of decision-making in a delay-discounting task, in which participants are required to decide between a larger/later and smaller/sooner reward (Dshemuchadse et al., 2013). In this study, participants were required to indicate which reward option (smaller/sooner or larger/later) they prefer by clicking an appropriate button with the mouse cursor (e.g., selecting a $10 reward right now or a $100 reward a week later). The starting position of the cursor was fixed in each trial and the participant had to move the cursor from the initial position to the end position (i.e., the response box of their choice). The researchers measured the degree of deviation of the cursor from the straight line linking the start and the end positions. They found that cursor trajectories tend to veer toward a smaller/sooner option even though the final choice was in favor of the larger/later option. According to the authors, this deviation reflects the process of overcoming the attraction of competing rewards. Although this study was performed on a non-clinical sample, its results provide valuable methodological insight into the nature of behavioral disinhibition. Since delay discounting (overwhelming preference for smaller/sooner rewards over larger/later) was found to be representative of an individual’s trait impulsivity (Gu, 2012), it is reasonable to suggest that trajectories of the cursor in choice reaching is likely to provide insight into the behavioral disinhibition when applied to a different measure of disinhibition such as a go/No-go task as well.

Another reason in favor of mouse cursor trajectory as an enhancement tool lies in the significance of motor control in ADHD. Evidence suggests that children with ADHD often lack fine motor control. For example, Tseng et al. (2004) demonstrated that among children with ADHD more than a half of variance in fine motor skills, such as dexterity (ability to use small muscles to manipulate fingers) and hand-eye coordination, is accounted by attention and impulse control. These skills were accessed through the use of Bruininks-Oseretsky Test of Motor Proficiency (BOTMP) which measures precision, coordination and speed of hand movements (Fine, 1979). In line with these findings, children with ADHD were found to have more coordination problems than healthy controls (Fliers et al., 2008). These problems included manual dexterity as well as aiming and catching as assessed with ball task.

Indirect evidence for motor control as an indication of ADHD can be found in the fact that performance on the Kaufman Hand Movement task, a form of continuous motor assessment, was found among the few associated with rating scales and quality-of-life measurements (Barkley and Fischer, 2011). Together, these findings imply that the continuous motor measure is likely to serve to improve the ecological validity of performance-based EF-tests in general and ADHD in particular.

In summary, these findings point to the hypothesis that individual differences pertaining to impulsivity and behavioral inhibition can be reflected in motor behavior. To test this hypothesis, we chose one of the most common measures of disinhibition, the go/No-go task. Performance in this task has been found to be well associated with trait impulsivity, as well as impulsivity-related disorders like alcohol abuse (Marczinski and Fillmore, 2003), cocaine abuse (Fillmore and Rush, 2006), or pathological gambling (Kertzman et al., 2008).

In a typical go/No-go paradigm, in each trial, participants are presented with one of two types of stimuli: “go,” which requires participants to respond by pressing a button, or “No-go,” which requires participants not to respond. Participants’ performance is evaluated based on their response time and accuracy (Georgiou and Essau, 2011). In our study, we compared the traditional version of this task in which participants responded by pressing the key, to the augmented version. In our augmented version of the go/No-go task, participants had to reach for the key drawn on the screen with the mouse cursor (from the uniform starting location) to make a response. Performance in both versions was evaluated in association with an individual’s ADHD profile.

**FIGURE 1:**
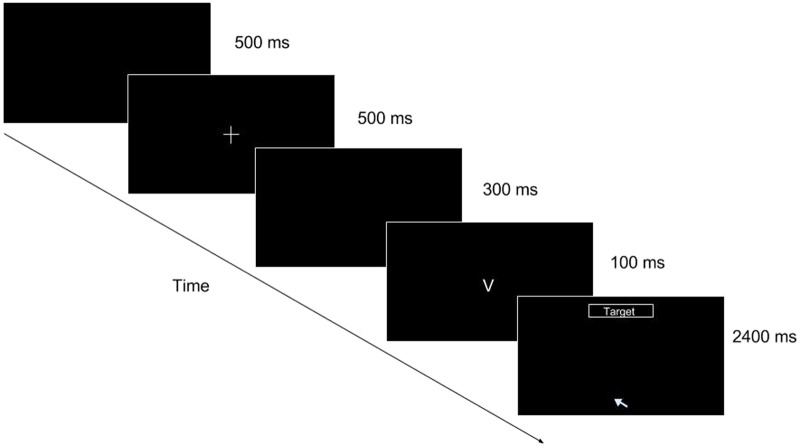
Go/No-go task (mouse movement condition single trial illustration). As the trial progresses, the participant is shown a blank screen for 500 ms, followed by a fixation cross for 500 ms, followed by another blank for 300 ms. After the second blank, a stimulus appears for 100 ms. At the offset of the stimulus (when “V” or “2” disappears), the target box appears at the top of the screen and the cursor is placed at the bottom center of the screen, prompting the participant to make a response by reaching for it with the mouse cursor.

We used a modified version of go/No-go task (**Figure 1**) used by Gorman Bozorgpour et al. (2013), with the same stimuli to inhibit and elicit a response. We investigated the extent to which performance measures such as response time, accuracy, and mouse-specific measures are associated with Conners Adult ADHD rating scale (Conners et al., 1999b).

In the keypress condition, response time and the standard deviation of response time (both in go- and No-go trials) served as the main predictor variables. In the mouse movement condition, movement-specific measures—mean maximum acceleration, velocity, and total distance of mouse motion served as the predictor variables.

These variables were selected as predictors based on the previous research in hand movement among children with ADHD (Eliasson et al., 2004), which found significant differences in peak acceleration and path length ratio (ratio between the distance traveled by a participant and distance between start and end point) between children with ADHD and healthy controls. On average, children with ADHD had higher peak acceleration and larger path length ratio. Velocity, while not differing significantly between ADHD and healthy controls, had higher intra-individual variability among individuals with ADHD.

Additional reason for the choice of velocity and velocity-based measures for ADHD assessment can be found in the eye-tracking studies on ADHD populations. These studies (Munoz et al., 2003; Rommelse et al., 2008) point to the specific deficits ADHD individuals have in saccadic control, a measure characterized by the peak velocity of eye movement. Given the coordination between hand and eye movements in goal-directed tasks (Binsted et al., 2001) it is reasonable to assume that the peak velocity – “saccadic” hand movement – will be indicative of ADHD in our experiment as well.

It should be noted that this study focuses on a healthy student sample; thus implications of the current study in resolving issues relevant to clinical practice are limited. However, individuals with sub-threshold ADHD (i.e., those experiencing symptoms but not meeting full criteria for disorders) still have an elevated risk of other psychological problems such as addiction, as compared to healthy controls (Hong et al., 2014; Norén Selinus et al., 2016). For this reason, we think that studying a healthy student sample is worthwhile. Furthermore, as Faraone et al. (2006a,b) point out, individuals who do not meet full clinical criteria might have a milder form of ADHD. According to the authors, some DSM-IV criteria for ADHD diagnosis are too stringent, potentially excluding individuals with late-onset ADHD and depraving them from proper treatment. These individuals, however, still experience negative ADHD symptoms. For this reason, we think that studying behavior of healthy college students with elevated ADHD scores is important as an analog of the sub-threshold population.

## Materials and Methods

### Participants

A total of 230 Texas A&M 1st- or 2nd-year undergraduate students who enrolled in an introductory psychology course participated in the experiment for course credit. They were randomly assigned to one of two conditions—the keypress (*n* = 125) or the mouse movement condition (*n* = 103). Of these participants, 14 participants did not complete the entire experiment. Thus, the data from 116 participants in the keypress condition (female = 87, male = 29) and 100 participants in the mouse movement condition (female = 72, male = 28) were analyzed. From these participants, we removed the data from one participant in the keypress condition and 11 participants in the mouse movement condition as their accuracy for the go/No-go task was below 50%. We reasoned that these participants did not understand the instructions or did not do the experiment as instructed. Thus, the data from 115 participants in the keypress condition (females = 86, males = 29) and the data from 89 participants (females = 62, males = 27) in the mouse movement condition were analyzed. In the keypress condition, mean age was 19.76 (*SD* = 1.16), while in mouse condition mean age was 19.62(*SD* = 1.18). In the keypress condition, mean ages were 19.7 (*SD* = 1.21) years for females and 19.8 (*SD* = 1.03) for males. In the mouse movement condition, the mean age for females was 19.5 (*SD* = 1.09) years and that for male was 19.7 (*SD* = 1.38) years. **Tables 1**, **2** summarize means and standard deviations of scores on each CAARS subscale, as well as the number of participants with t-scores above the threshold (65).

**Table 1:** Descriptive statistics for CAARS scores in keypress condition.

	A (Inattention)	B (Hyperactivity)	C (Impulsivity)	D (Self-concept)	E (Inattentive)	F (impulsive)	G (Total)	H (ADHD index)
Mean	51.02	45.26	48.90	45.37	59.53	52.31	57.40	50.95
*SD*	7.80	7.61	8.05	6.64	9.06	10.36	10.34	8.82
N above threshold	7	1	3	1	35	16	27	11

*N above threshold indicates number of participants whose t-score on a given subscale exceeds the cut-off of 65, indicating elevated symptom severity (Solanto et al., 2012).*

**Table 2:** Descriptive statistics for CAARS scores in mouse movement condition.

	A (Inattention)	B (Hyperactivity)	C (Impulsivity)	D (Self-Concept)	E (Inattentive)	F (impulsive)	G (Total)	H (ADHD index)
Mean	52.16	51.53	47.81	49.58	56.73	51.11	55.16	52.09
*SD*	8.40	8.97	9.22	8.93	10.05	10.32	10.61	8.58
N above threshold	8	7	3	6	18	8	15	9

*N above threshold indicates number of participants whose t-score on a given subscale exceeds the cut-off of 65, indicating elevated symptom severity (Solanto et al., 2012).*

This study was carried out in accordance with the recommendations of Texas A&M University Institutional Review Board. The protocol was approved by Texas A&M University Institutional Review Board. All participants gave written informed consent in accordance with the Declaration of Helsinki.

### Procedure

Participants completed the following tasks in sequence:

1. Go/No-go task
2. Conners Adult ADHD questionnaire (CAARS)

The protocol was as follows. First, participants performed a go/No-go task in one of the two conditions—keypress or mouse movement. In the keypress condition, participants were instructed to press the space bar on their keyboard when presented with the “go” cue (i.e., letter “V”). They were also instructed not to press anything when presented with the “No-go” cue (i.e., digit “2”). The mouse movement condition was identical to that of the keypress condition. However, the participants in the mouse movement condition were instructed to click a button on the screen with their computer mouse. At the completion of the go/No-go task (220 trials in total), participants answered the CAARS questionnaire (Macey, 2003). The go/No-go task consisted of 220 trials; among these, the first 20 trials were practice trials. Each trial started with a blank screen for 500 ms, followed by a fixation cross for another 500 ms. After the fixation cross, another blank screen followed for 300 ms. After the second blank screen, a “go” or “No-go” stimulus (“V” for go and “2” for No-go) was presented for 100 ms. The stimuli were displayed in a white font on a black background. The stimuli were either the letter “V” (go stimulus) or the number “2” (No-go stimulus). Out of 200 trials, 100 were go trials (50%) and the remaining 100 were No-go trials (50%). Either the “go” or “No-go” stimulus was followed by another blank for 2400 ms.

With the exception that participants were instructed to indicate their response at the top-center of the screen for the mouse movement condition, the two conditions are identical. To monitor the trajectory of mouse movement, the cursor was placed at (0, -280) at the offset of stimulus presentation [where (0,0) represents the center of the screen]. Our program traced *x*–*y* coordinate locations of the moving cursor every 10 ms. In both the keypress and mouse movement conditions, we analyzed omission and commission errors as well as response times for go- and No-go trials. Omission error occurs when a participant does not make a response to the “go” stimulus when they should (in this experiment, letter “V”). Conversely, commission error, in this case, means providing a reaction to a “no-go” stimulus (number “2”). In the keypress condition, measurement began at the onset of stimulus presentation. In the mouse movement condition, response collection started at the offset of stimulus presentation.

#### Conners’ Adult ADHD Questionnaire

The Conners Adult ADHD questionnaire – self-report long version (CAARS-S: L) is a widely accepted ADHD assessment tool. This 66-item measure asks participants to indicate how accurate are the statements from the questionnaire in their description of participants’ personal feelings currently and from the past 2 weeks (Conners et al., 1999a,b). Responses are coded on a scale of 0–3, such that higher scores represent a statement’s stronger indication of a participants’ current condition. Internal consistency estimates for CAARS range from 0.79 to 0.90 for all subscales in the total sample. Validity estimates fall in a range from 0.82 to 0.93 for all subscales in the total sample.

Scores on CAARS are mapped onto eight subscales. Higher scores on subscale A (Inattention/Memory problems) indicate troubles with concentration, planning, completing tasks, forgetfulness, and absent-mindedness above the norm, and disorganization. Subscale B (Hyperactivity/Restlessness) serves as an indicator of problems with fidgeting, staying on the same task for long periods of time, and overall restlessness. C (Impulsivity/Emotional lability) indicates low frustration tolerance, mood changes, being easily angered or irritated, and an overall inclination to engage in more impulsive acts than others. Higher scores on subscale D (Problems with self-concept) are indicative of lower self-esteem and self-confidence, as well as poor social relationships. DSM-IV subscales, namely, E (DSM-IV: Inattentive symptoms), F (DSM-IV: Hyperactive-Impulsive Symptoms), G (DSM-IV: ADHD symptoms total), reflect behavior consistent with that described by Inattentive, Hyperactive-Impulsive and Combined subtypes of ADHD, respectively. Finally, subscale H (ADHD index) serves as a general indication of whether a person should be considered at risk for ADHD. Individual scores obtained for these subscales served as dependent variables for the experiment.

### Design

The experiment was designed with one between-subjects factor (keypress, mouse movement conditions). We selected to employ the between-subjects design rather than a within-subjects design to avoid potential instructional confusion and fatigue (a within-subjects design would increase the number of trials for an individual to 400).

**FIGURE 2:**
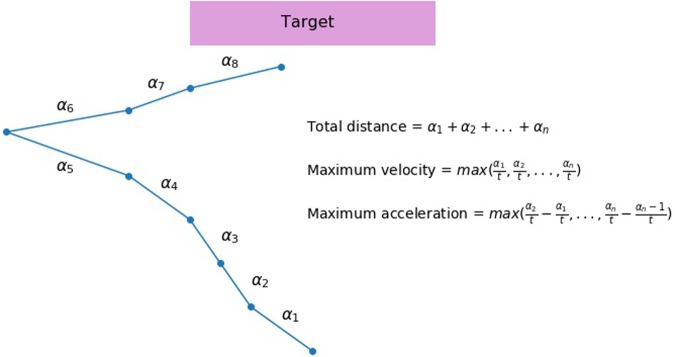
An illustration of a hypothetical mouse trajectory in a no-go trial. This hypothetical trial had ended before participant had made a response (i.e., clicking the button). Total distance (measured by pixels) of a mouse movement is calculated as a sum of all distances from the starting position to the end position that are recorded at each time slice (α_1_, α_2_ … α_n_). If 2400 ms pass before the participant makes a response, all trajectories recorded during the trial are added. No movement is treated as movement with distance equal to zero. Velocity is calculated as a length of distance between two sets of coordinates divided by the time elapsed between their recordings (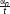, t - constant). A maximum velocity is selected for each trial. Acceleration is calculated as an increase in velocity between two recordings, and then a maximum value for each trial is selected.

In the keypress condition, main independent variables were performance accuracy, response time and standard deviation of response time, separately in go- and No-go trials. In the mouse movement condition, main independent variables include mean maximum acceleration, velocity, and total distance. We also measured response time and accuracy to compare the mouse movement and key press conditions directly. Total distance represents mean of total Euclidean distance from the original starting position of the cursor to the position of the cursor at the end of the trial. Each trajectory was broken down into adjacent sets of coordinates according to the time of their recording. An example of such sets comprising a full trajectory in a hypothetical trial can be seen in the **Figure 2**. If 2400 ms passed and the trial ended with no response, all trajectories recorded during the trial were added (no movement was treated as movement with distance equal to zero). All trajectories were time-normalized. Velocity was calculated as the mean maximum speed with which movement between two adjacent sets of coordinates took place. Mean maximum acceleration stands for change in velocity of the mouse movement between the two adjacent parts of the trajectory. Because these measures were highly skewed, all independent measures were log-transformed.

#### Intra-Subject Variability

Recent studies in ADHD assessment (Hauser et al., 2016) point to the intra-subject variability of response measures, such as response time, to be one of the best indicators of ADHD. To access the intra-subject variability of our measures, we have calculated a variability coefficient for each measure. It was calculated as a ratio between standard deviation and mean value of a certain measure for each participant. Variability coefficient was calculated for go and No-go trials separately. We have investigated the correlations between the measures of intra-subject variability (variability coefficient, VC) and CAARS scores.

### Apparatus

Keypress sessions were implemented using PsychoPy software environment (Peirce, 2009). The mouse cursor tracking sessions of the experiment were implemented using the OpenSesame software environment (Mathôt et al., 2012) with the plug-in “mousetrap” for mouse cursor movement tracking (Kieslich and Henninger, 2017). We used six desktop computers (HP de 7900 systems with an E8400 Core 2 Duo 3.0 GHZ processor) and monitors (19-inch wide flat panel display; HP L1908wi) for data collection. All participants used the same Dell Optical Mouse with USB connection (Dell 0C8639 USB 2 Button Scrollwheel Optical Mouse). The pointer speed of the mice was set as medium and the resolution of the monitor was fixed as 1,440 × 900.

## Results

To scrutinize the effect of our enhancements of the go/No-go task, we conducted a number of statistical analyses. To make sure the results in our experiment are comparable, we first examined accuracy and response time in keypress and mouse motion conditions. After that we compared high and low ADHD groups using *t*-tests to evaluate the discriminatory ability of keypress and mouse measures. To evaluate the explanatory ability of mouse and keypress measures, we explored the correlations between different keypress or mouse measures on one hand and CAARS scores on the other. To illustrate the role of response variability and compare the predictive power of variability in keypress and mouse measures, we investigated the correlations between variability coefficients in both conditions and CAARS scores. Additionally, to illustrate the skewness of reaction times (both in keypress and mouse motion conditions), we fit the reaction times in both conditions to ex-Gaussian distribution and explored their correlations with questionnaire scores. Finally, we performed step-wise regressions to identify relevant predictors that were most associated with ADHD ratings.

These separate measures of association help clarify how the key press and mouse movement conditions diverge. First, a non-parametric measure of correlation (Spearman’s rho) assumes no linearity in association between two variables; therefore this measure is relatively immune to outliners. The Pearson’s correlation measure assumes linearity between variables and it is notoriously vulnerable to outliers (i.e., a few outliers can inflate correlation measures). Second, the extreme group comparison with standard *t*-tests helps to separate the healthy and sub-threshold (as discussed before) groups, providing further insight into the disparity between ADHD rating scale and performance tests. Finally, stepwise regression (which assume linearity) served the purpose of identifying an independent and uncorrelated combination of mouse movement features associated with ADHD-related traits (such as impulsivity or inattentiveness) or subtypes of ADHD (such DSM-IV: primarily inattentive). Thus, these association measures, though related, provide more stringent and informative tests of association.

### Go/No-Go Task Performance

#### Accuracy in the Keypress and Mouse Movement Conditions

To make the traditional go/No-go task (keypress condition) and the augmented go/No-go task (mouse movement condition) compatible, the accuracy in the two conditions was kept at a ceiling level (**Table 3**). Accuracy in go and No-go trials, both in keypress and mouse movement conditions, were nearly identical to those found in the Gorman Bozorgpour et al. (2013) study and the Nieuwenhuis et al. (2003) study. Additionally, no difference in accuracy was observed between conditions in our experiment among go (*t*(185) = 1.46, *p* = 0.14, *d* = 0.21, 95% CI_d_ [-0.07, 0.48]), and no-go (*t*(198) = -1.88, *p* = 0.06, *d* = -0.25, 95% CI_d_ [-0.53, 0.02]) trials alike. This suggests that the difficulty in the two conditions was comparable.

**Table 3:** Accuracy in keypress and mouse movement conditions.

	Keypress		Mouse	
	Go	No-go	Go	No-go
Mean	0.99	0.96	0.97	0.99
*SD*	0.06	0.11	0.06	0.07

#### RT in Keypress and Mouse Movement Conditions

Mean response time in the mouse movement condition was significantly longer than the keypress condition (both in go and no-go trials) (**Table 4**). As expected, response time differed significantly between the keypress and mouse condition, both in the go (*t*(98) = -29.37, *p* < 0.001, *d* = -4.63, 95% CI_d_ [-5.16, -4.10]) and no-go (*t*(27) = -26.65, *p* < 0.001, *d* = 5.78, 95% CI_d_ [4.82, 6.74]) trials.

**Table 4:** Response time for keypress and mouse movement conditions (ms).

	Keypress		Mouse	
	Go	No-go	Go	No-go
Mean	382.28	432.07	1049.65	2286.79
*SD*	58.01	333.91	208.22	237.19

### Go/No-Go Task Performance and ADHD Questionnaire Scores

#### Discriminating Between High and Low ADHD Populations

To evaluate the extent to which keypress and mouse movement measures capture differences in ADHD and disinhibition traits between sub-threshold and normal populations, we separated our participants into two groups: high (those in or above the 75th percentile of CAARS subscales) and low (those in or below the 25th percentile of CAARS subscales). We then compared these two groups with respect to their performance measures.

Given the keypress condition, we found no significant differences in response time and accuracy measures between low and high ADHD groups; *t*’s < 1.48, *p*’s > 0.14.

**FIGURE 3:**
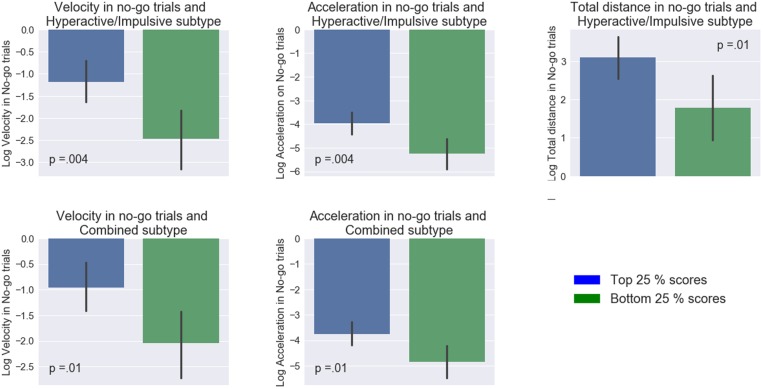
Comparisons between top and bottom quartiles of ADHD subtype inclination scores among different measures. On the *x*-axis, blue and green color bars represent low and high (25th and 75th percentiles of ADHD scores). Mapped on the *y*-axis, are the log-transformed values of the mean maximum velocity, mean maximum acceleration, and mean total distance. Error bars represent 95% confidence interval.

In contrast, the mouse movement condition showed a number of significant differences between the groups (**Figure 3**). Specifically, the high and low groups in the Hyperactive/Impulsive subscale were significantly different in their mean maximum acceleration (*t*(42) = 3.03, *p* = 0.004, *d* = 0.91, 95% CI_d_ [1.55, 0.27]), velocity (*t*(42) = 3.04, *p* = 0.004, *d* = 0.92, 95% CI_d_ [1.55, 0.27]) and total distance (*t*(42) = 2.57, *p* = 0.01, *d* = 0.78, 95% CI_d_ [1.40, 0.14]) in no-go trials. The high-low groups in Combined ADHD subtype were also significantly different in their mean maximum acceleration (*t*(42) = 2.57, *p* = 0.01, *d* = 0.79, 95% CI_d_ [1.42, 0.15]) and velocity in no-go trials (*t*(42) = 2.62, *p* = 0.01, *d* = 0.78, 95% CI_d_ [1.40, 0.14]). Similarly to the keypress condition, no difference in accuracy between high and low ADHD groups was found: *t*’s < 0.84, *p*’s > 0.06.

These results imply that participants’ ADHD profiles were most strongly associated with performance in the augmented go/No-go task as compared to the keypress version of this task. Individuals with a stronger inclination toward aforementioned subtypes had higher acceleration as well as mean peak velocity.

#### Correlations Between Go/No-Go Performance and Questionnaire-Based ADHD Measurement

We analyzed the correlation between performance in different conditions of go/No-go task and ADHD/disinhibition profile. **Tables 5**, **6** summarize the results of Spearman’s rank-order correlations between behavioral measures (keypress and mouse movement, respectively) and scores on CAARS subscales. We selected Spearman’s rank correlation because this correlation measure is less susceptible to outliers and makes no assumption of linearity.

**Table 5:** Spearman’s correlations between log-transformed keypress measures and log-transformed CAARS scores.

	Impulsivity/Emotional lability	Problems with self-concept	DSM-IV: Impulsive	DSM-IV: Inattentive	DSM-IV: ADHD symptoms total
Mean RT in no-go	-0.055	0.069	-0.044	-0.054	-0.044
SD RT in no-go	0.037	0.21	0.047	0.068	0.049
Accuracy in no-go	0.092	-0.003	0.095	0.068	0.078
Mean RT in go	-0.041	0.01	-0.025	-0.058	-0.045
SD RT in go	0.005	0.106	0.004	0.0002	0.017
Accuracy in go	-0.001	-0.2*	-0.02	-0.049	-0.043

*^∗^p ≤ 0.05, ^∗∗^p ≤ 0.001.*

**Table 6:** Spearman’s correlations between log-transformed mouse movement measures and log-transformed CAARS scores.

	Impulsivity/emotional lability	Problems with self-concept	DSM-IV: Hyperactive-Impulsive Symptoms	DSM-IV: Inattentive symptoms	DSM-IV: ADHD symptoms total
Velocity in go trials	0.204	0.11	0.105	0.085	0.104
Velocity in No-go trials	**0.328^∗∗^**	0.13	0.237*	0.22*	0.227*
Acceleration in go trials	0.212^∗^	0.121	0.11	0.1	0.114
Acceleration in No-go trials	**0.33^∗∗^**	0.12	0.238*	0.23*	0.235*
Total distance in go trials	0.123	0.118	0.149	0.047	0.083
Total distance in No-go trials	**0.305^∗∗^**	0.12	0.217*	0.183	0.198

*^∗^p ≤ 0.05, ^∗∗^p ≤ 0.01. The scores with bold type face represent statistically significant correlations after controlling the false discovery rate at q = 0.05 (Benjamini and Hochberg, 1995). To make sure these correlations hold for “non-clinical” participants, we reanalyzed Spearman’s rank correlation after removing participants whose CAARS scores exceeded the threshold level specified in **Table 2**. This additional analysis indicates that correlation levels without above-threshold participants were by and large analogous to those reported in this table (see Supplementary Material for this analysis).*

**FIGURE 4:**
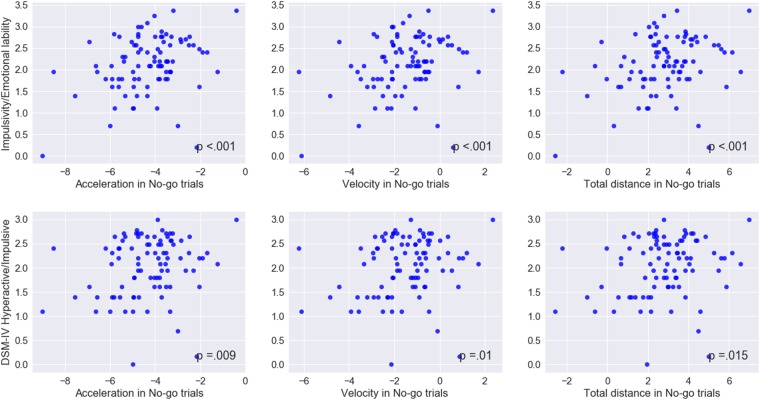
Correlations between mouse movement and impulsivity measures. Mapped on the *x*-axis, are log-transformed values of the mean maximum acceleration, mean maximum velocity and mean total distance. Shown on the *y*-axis, are log-transformed values of the scores on trait Impulsivity/Emotional lability and inclination toward Hyperactive/Impulsive subtype of ADHD scales.

Among the keypress measures (**Table 5**), a significant association was observed only between accuracy in go trials and problems with the self-concept subscale specified in CAARS (ρ = -0.2, *p* = 0.03). Among mouse movement measures, velocity and total distance in no-go trials, as well as acceleration in go- and no-go trials were significantly correlated with indicators of trait impulsivity (subscale C – Impulsivity/Emotional lability) and inclination toward Hyperactive/Impulsive subtype of ADHD (subscale F). Mouse movement measures also turned out to have significant relationships with primarily Inattentive (subscale E) and Combined (subscale G – ADHD symptoms total) subtypes of ADHD. **Figure 4** illustrates correlation between log-transformed scores on trait-impulsivity and inclination toward Hyperactive/Impulsive subtype of ADHD on the *y*-axis and log-transformed mouse measures on *x*-axis.

These results indicate that mouse movement measures were strongly associated with trait disinhibition and ADHD symptom inclination, as compared to performance in traditional keypress version of the go/No-go task.

Although these levels of correlation might appear low, a meta-analysis of studies of associations between self- or other-ratings of EF and performance in neuropsychological tests has shown that most of the studies reported correlation coefficients from 0.0 to 0.3 (Toplak et al., 2013). As case in point, the majority of correlations in the study performed by Miranda et al. (2015) were in range from 0.1 to 0.3. Considering this, the correlation levels in this study are quite high.

#### Within-Subject Variability Measures and ADHD

No significant correlation was observed between keypress variability coefficients and any of the questionnaire-based ADHD measures. In contrast, as seen in **Table 7**, mouse movement measures had a number of significant correlations. Variability in acceleration in go trials emerged as a feature associated with the largest number of facets of ADHD profile, such as Hyperactivity/Restlessness, Inattention/Memory problems and inclination toward different subtypes of ADHD. Variability in velocity was found to be associated with Hyperactivity/Restlessness, as well as inclination toward Hyperactive/Impulsive and Combined subtypes of ADHD. Finally, variability in total distance in go had significant correlations with inclination toward primarily Hyperactive/Impulsive subtype of ADHD and ADHD index, the indication of overall clinical severity of a participant’s ADHD profile. No significant relationship was observed between variability in different mouse movement in No-go trials and any of the CAARS subscale scores.

**Table 7:** Spearman’s correlations between variability coefficients (VC) of mouse movement measures and log-transformed CAARS scores.

	Inattention/Memory problems	Hyperactivity/Restlessness	DSM-IV: Inattentive symptoms	DSM-IV: Hyperactive-Impulsive Symptoms	DSM-IV: ADHD symptoms total	ADHD index
Velocity VC in go trials	0.188	0.338**	0.181	0.291**	0.236*	0.14
Acceleration VC in go trials	0.283**	0.293**	0.262*	0.264*	0.267*	0.165
Total distance VC in go trials	0.123	0.254*	0.172	0.243*	0.205*	0.239*

*^∗^p ≤ 0.05, ^∗∗^p ≤ 0.001.*

We have also investigated the variability in reaction time distributions in both conditions. Specifically, we selected the p parameters of ex-Gaussian reaction time distributions. Parameters of this distribution are known to be representative of implicit and explicit cognitive processes involved in reaction to the stimuli (Ratcliff, 1978; Matzke and Wagenmakers, 2009). These parameters, – μ, σ, and τ – represent mean and standard deviation of the Gaussian component and the mean of the exponential component, respectively. Among these parameters, μ was found to be indicative of attentional cognitive processes, primarily stimulus-driven and non-analytic, and correlated with duration of residual processes, such as sensory or motor processing. Parameter τ, on the other hand, is considered to be associated with duration strategic processes and reflects the decisional part of RT (Rotello and Zeng, 2008).

With regard to these findings, we fitted the ex-Gaussian distribution to every subject’s responses and estimated the parameters for every individual. We correlated these parameters using Spearman’s correlation with scores on different ADHD subscales. No significant correlation was observed in both keypress and mouse movement condition (for all measures, ρ’s < 0.17, *p*’s > 0.11).

#### Step-Wise Regression

To investigate the explanatory ability of keypress and mouse measures, we applied stepwise regression analysis separately to the key press and mouse movement conditions. We used CAARS subscales as dependent variables and other keypress and mouse movement variables as predictors. Stepwise regression, although it assumes linearity, identifies a unique and uncorrelated combination of mouse movement features associated with ADHD-related traits or subtypes of ADHD. Final models were selected using the Akaike Information Criterion (AIC).

In the keypress condition, significant relationships were observed between SD RT in no-go trials and the self-concept subscale [*B* = 0.078, *t*(51) = 2.75, *p* < 0.001], explaining 13% of variance [*F*(1,51) = 7.6, *p* = 0.008, *R*^2^ = 0.13, *R*^2^_adj_ = 0.11]. Inclination toward Inattentive subtype was significantly predicted by mean RT in go trials [*B* = -0.85, *t*(50) = 2.54, *p* = 0.012] and SD RT in No-go trials [*B* = 0.03, *t*(50) = 2.03, *p* = 0.04], explaining 16% of variance [*F*(2,50) = 4.86, *p* = 0.011, *R*^2^ = 0.16, *R*^2^_adj_ = 0.13]. Finally, inclination toward Combined subtype had a marginally significant association with mean RT in go trials [*B* = -1.01, *t*(50) = 2.4, *p* = 0.02], explaining 11% of variance [*F*(2,50) = 3.07, *p* = 0.05, *R*^2^ = 0.11, *R*^2^_adj_ = 0.07]. **Table 8** summarizes all models selected using AIC in keypress condition.

**Table 8:** Keypress measures regressions (AIC).

	*R*^2^	Adj. *R*^2^	Mean RT in No-go trials	SD RT in No-go trials	Accuracy in No-go trials	Mean RT in go trials	SD RT in go trials	Accuracy in go trials
Inattention/Memory problems	0.06	0.03	0.19			-0.77		
Hyperactivity/Restlessness	0.09	0.05	0.3	.		-1.04		
Impulsivity/Emotional lability	0.05	0.03		0.04				
Problems with self-concept	0.13	0.11		0.07^∗∗^				
DSM-IV: Inattentive symptoms	0.16	0.13		0.03^∗^		-0.85^∗^		
DSM-IV: Hyperactive/Impulsive symptoms								
DSM-IV: ADHD symptoms total	0.11	0.07	0.19			-1.01^∗^		
ADHD index								

*^∗^p ≤ 0.05, ^∗∗^p ≤ 0.01, ^∗∗∗^p ≤ 0.001.*

In the mouse movement condition, the results indicate the acceleration in no-go trials to have a significant relationship with both impulsivity measures – Impulsivity/Emotional lability [*B* = 0.167, *t*(86) = 4.01, *p* < 0.001], explaining 19% of variance [*F*(1,86) = 16.05, *p* < 0.001, *R*^2^ = 0.19, *R*^2^_adj_ = 0.15] and DSM-IV: Hyperactive/Impulsive symptoms [*B* = 0.111, *t*(87) = 2.68, *p* = 0.009], explaining 8% of variance [*F*(1,87) = 7.19, *p* = 0.009, *R*^2^ = 0.08, *R*^2^_adj_ = 0.07]. Acceleration in no-go trials has also been found to be indicative of overall gravity of ADHD symptoms –ADHD index [*B* = 0.065, *t*(87) = 2.02, *p* < 0.05]. Acceleration in no-go trials explained only 5% of variance in ADHD index scores [*F*(1,87) = 4.08, *p* < 0.05, *R*^2^ = 0.05, *R*^2^_adj_ = 0.03].

Inattention and Memory problems subscale was significantly related to total distance in No-go trials [*B* = -0.28, *t*(86) = 2.16, *p* = 0.03] and acceleration in no-go trials [*B* = 0.36, *t*(86) = 2.28, *p* = 0.02]. This model was marginally significant and explained 6% of variance [*F*(2,86) = 2.6, *p* = 0.07, *R*^2^= 0.06, *R*^2^_adj_ = 0.03]. **Table 9** summarizes models selected using AIC in mouse movement condition.

**Table 9:** Mouse movement measures regressions (AIC).

	*R*^2^	Adj. *R*^2^	Acceleration in No-go trials	Velocity in No-go trials	Total distance in No-go trials	Acceleration in go trials	Velocity in go trials	Total distance in go trials
Inattention/Memory problems	0.06	0.03	0.36*		-0.29*			
Hyperactivity/Restlessness								
Impulsivity/Emotional lability	0.19	0.15	0.14***			3.53	-3.51	
Problems with self-concept	0.05	0.02				-3.96	4.1	
DSM-IV: Inattentive symptoms	0.13	0.08	0.31		-0.21	3.74	-3.98	
DSM-IV: Hyperactive/Impulsive symptoms	0.08	0.07	0.11**					
DSM-IV: ADHD symptoms total	0.07	0.04	0.3		-0.2			
ADHD index	0.05	0.03	0.06*					

*^∗^p ≤ 0.05, ^∗∗^p ≤ 0.01, ^∗∗∗^p ≤ 0.001.*

These findings point to stronger associations between mouse movement measures – namely, acceleration – and different facets of ADHD profile, compared to the keypress measures. Acceleration describes greater portion of variance in CAARS scores and is associated with a greater number of subscales.

## Discussion

Performance-based measures of EF such as go/No-go tests have served as an initial tool to probe individuals’ vulnerability to ADHD (Epstein et al., 2003). The problem, however, is that these tests often fail to converge with rating scales such as Conner’s adult ADHD questionnaire (CAARS). How does this happen? Part of this discrepancy, we surmised, is due to the paucity of metric; traditional go/No-go tests take individual differences in accuracy and response time as indicators of variations in EF. However, these measures provide only two data points—binary correct/incorrect response and its latency; from these outcome measures alone, it is extremely difficult to infer the *process* by which an individual interacts with a judgment (e.g., “no go”) and action (e.g., not pressing a key). We hypothesized that this process can be reflected in motor behavior in navigating the computer mouse, and, by augmenting a standard go/No-go task with mouse-cursor movement measures, the discrepancy between rating scales and performance-based measures should be reduced.

Consistent with this prediction, we found that the augmented version of go/No-go task aligns well with ADHD rating scales, while traditional accuracy and response time measures, as collected in the keypress condition, continues to show discrepancy. Results show that speed and acceleration of cursor movement, as well as variability in these features, are well associated with different ADHD subtypes indicated by CAARS, while traditional measures of response time and accuracy reveal virtually no association. Moreover, mouse movement features have significant associations with inclination with both primarily Hyperactive/Impulsive and Combined subtypes of ADHD; while response time and accuracy measures obtained in the keypress condition lack significant correlations, variability coefficients in mouse movement measures had strong and multiple correlations with ADHD rating scores, making a solid case for them as a measure of ADHD, in line with previous studies (Eliasson et al., 2004). Together, these findings indicate that individual differences in impulsivity and behavioral inhibition can be well monitored by motor variables, such as maximum velocity and acceleration in reaching behavior. We suggest that traditional go/No-go tests can be augmented by mouse motion measures and part of the discrepancy between rating and performance measures of ADHD can be reduced significantly with these measures. It should be noted, however, that other factors might be influencing the variability in rating scale-based measures, as evidenced by the percentage of variance in ADHD scores explained by the predictor variables.

Decision making and motor control share a common utility of incorporating costs (e.g., metabolic expenditure, effort, and duration) and rewards (e.g., success in action pursuit) (Shadmehr et al., 2016). This incorporation of costs and rewards is subject to individual differences as individuals differ in their perception of uncertainty, neural noise, and decision biases (Körding and Wolpert, 2004; Wolpert and Landy, 2012; Morel et al., 2017). Successes of “stop” and “go” processes in decision making are driven by a utility function that integrates the costs of adopting different cognitive and sensorimotor strategies. In this model, an individual repeatedly accesses his options in each trial to make an “optimal decision” by assessing the information processed in the current trial and previous trials (Shenoy and Yu, 2011). We think that the variability of executive functioning in ADHD is manifested in this interactive process and dynamic motor measures such as mouse movement provide a window to capture this dynamic interaction and its variability in individuals.

Our findings also highlight the significance of using rating scale-based measures and performance-based (behavioral) tests jointly. Since many rating scales are theoretically and empirically based on one another [for example, a popular measure BDEFS was based off of the BDEFS-CA (Allee-Smith et al., 2013)], different rating scales are often inter-correlated by design. Moreover, the subjective interpretation of the questionnaire-based assessment is not always consistent. For example, one individual’s self-report of “severe” intensity may be another individual’s “minor.” As Hsu et al. (2017) argue, those with sub-threshold ADHD symptoms experience “higher mental effort and discomfort” than completely healthy individuals. In this regard, it is critical to apply rating scale-based measures and performance-based (behavioral) tests jointly. By augmenting traditional go/No-go tests with motor measures, a new performance-based test can be applied not only to the assessment of veridical ADHD, but also to the detection of feigning ADHD – behavior that goes completely undetected by the common questionnaire-based measures of this disorder (Rios and Morey, 2013).

It should be noted that as ADHD progresses differently in every individual as they age, tools should be able to account for those changes without relying on one’s memory. In a study that examined EF in aging adults, performance-based measures often fare better than questionnaire-based measures among older adults (McAlister and Schmitter-Edgecombe, 2016). Performance-based measures need to be developed toward more life-like situations to create a greater ecological validity. With technology developing and its relevance becoming more pronounced in everyday life, mouse-cursor tracking provides a sound method to examine the fluidity in the acquisition of a goal, executed in an apparatus used daily and globally.

One important limitation to our study is the source from which we recruited our participants. Our participants were recruited from a college student population, which might eliminate individuals at risk or experiencing severe ADHD. The college student population also may be more aware of the disorder and those affected may have already taken the proper steps to cope with EF impairments. With these considerations, it is imperative to increase the size and variety of our sample size in future studies in order to give a more accurate representation of the general population.

## Conclusion

Collectively, our results demonstrate a strong association between mouse movement properties and behavioral disinhibition. These results indicate that motor control and higher-order cognitive processes involved in goal planning and staying on task might share a common mechanism. We suggest that traditional performance tests relying exclusively on accuracy and response time can be improved by incorporating dynamic motor measures and in doing so, the discrepancy between rating scales and performance tests can be curtailed considerably.

## Author Contributions

AL, SS, and TY contributed conception and design of the study. AL programmed the experiments and performed the statistical analysis. AL, MW, and TY wrote and revised sections of the manuscript. All authors read and approved the submitted version.

## Conflict of Interest Statement

The authors declare that the research was conducted in the absence of any commercial or financial relationships that could be construed as a potential conflict of interest.
