# Supplementary material for: Augmented Go/No-Go Task: Mouse Cursor Motion Measures Improve ADHD Symptom Assessment in Healthy College Students
Source: Front Psychol. 2018 Apr 11;9:496. doi: 10.3389/fpsyg.2018.00496 (PMC5905239; doi:10.3389/fpsyg.2018.00496)
Supplement: Supplementary file 1 [file Data_Sheet_1.DOCX]

Supplementary Material

Augmented Go/No-go Task: Mouse Cursor Motion Measures Improve ADHD Symptom Assessment in Healthy College Students

Anton Leontyev, Stanley Sun, Mary Wolfe, Takashi Yamauchi^*^

*** Correspondence:** Takashi Yamauchi: takashi-yamauchi@tamu.edu

# Supplementary Data

## Distributions of gender, age, and CAARS scores in the key press and mouse movement conditions

To ensure our samples in the two conditions were comparable, we examined distributions of participants’ gender, age and CAARS scores. The chi-square test of proportions for independent samples showed no significant differences in the gender distributions in the two conditions; *X*^2^ (1, *N* =204) = .42, *p* = .51. For age, Kolmogorov-Smirnov test showed no difference in the age distributions of the two groups; *D* =.07, *p* = .97 For CAARS subscales, we found no differences in distributions in the keypress and mouse movement conditions in the subscales A, C, E, F, G and H; Kolmogorov-Smirnov test, for all comparisons, yielded *p*’s > .23. However, the distributions of subscales B and D were significantly different in the keypress and mouse movement conditions: the results of the Kolmogorov-Smirnov test were *p* < .001 on subscale B and *p* < .01 on subscale D (Supplementary Figure 1).

**Supplemental Figure 1. Distributions of CAARS scores.** P-values indicate the results of Kolmogorov-Smirnov test. Distributions are plotted using local regression (LOESS) smoothing algorithm. LOESS is a non-parametric method in which simple least squares regression models are fir to the localized subsets of data (Cleveland and Devlin, 1988).

Because the distributions of the subscale B and D were significantly different in the key press and mouse movement conditions, we applied a min-max transformation ($x_{transformed}=\frac{x_{n}-min(x)}{\max\left( x \right)-\min(x)}$, where *x* represents all scores on a subscale, *x_n_* is *n^th^* participant’s raw score and *x_transformed_* is *n^th^* participant’s transformed score). After this transformation, we compared two distributions, with Kolmogorov-Smirnov test yielding no significant differences, p = .10 on subscale B and p =.30 on subscale D.

**Supplemental Figure 2. Distributions of min-max-transformed scores**. P-values indicate the results of Kolmogorov-Smirnov test. Distributions are plotted using local regression (LOESS) smoothing algorithm.

With the transformed subscale scores, we re-applied the same analyses as described in the Result section. Because the min-max transformation is linear, Spearman’s rank-order correlation yielded the same results as shown in Tables 5 and 6. Results from the extreme group comparison were also analogous to those reported in the main text (t-tests, sec. 3.2.1). Given the keypress condition, there were no significant differences in response time and accuracy measures between low and high ADHD groups; *t*’s <0.75, *p*’s > .17. In the mouse movement condition, the high and low groups in the Hyperactive/Impulsive subscale were significantly different in their mean maximum acceleration (*t*(42) = 2.88, *p* =.006, *d* = -0.87, 95% CI_d_ [-1.51, -0.23]), velocity (*t*(42) = 2.97, *p* =.004, *d* = -0.89, 95% CI_d_ [-1.53, 0.25]) and total distance (*t*(42) = 2.78, *p* =.008, *d* = 0.83, 95% CI_d_ [-1.47, -0.20]) in no-go trials. The high-low groups in Combined ADHD subtype were also significantly different in their mean maximum acceleration (*t*(42) = -2.80, *p* =.007, *d* = -0.84, 95% CI_d_ [-1.48, -0.21]), velocity (*t*(42) = -2.82, *p* =.007, *d* = -0.85, 95% CI_d_ [-1.48, -0.22]) and total distance in no-go trials (*t*(42) = -2.57, *p* =.01, *d* = -0.77, 95% CI_d_ [-1.40, -0.14]).

Stepwise regression analysis also showed results analogous to those performed without the min-max transformation. In the keypress condition, significant relationships were observed between SD RT in no-go trials and the self-concept subscale (*B* = 0.03, *t*(51) = 2.76, *p* =.008), explaining 13 % of variance (*F*(1,51) = 7.6, *p* =.008, *R*^2^ =.13, *R*^2^_adj_ = .11). Inclination towards Inattentive subtype was significantly predicted by mean RT in go trials (*B* = -0.51, *t*(50) = -2.58, *p* =.01) and SD RT in No-go trials (*B* = 0.02, *t*(50) = 2.03, *p* =.047), explaining 16% of variance (*F*(2,50) = 4.87, *p* =.012, *R*^2^ =.16, *R*^2^_adj_ = .13). Finally, inclination towards Combined subtype had a marginally significant association with mean RT in go trials (*B* = -0.57, *t*(50) = -2.4, *p* = 0.02), explaining 11 % of variance (*F*(2,50) = 3.07, *p* =.05, *R*^2^ =.11, *R*^2^_adj_ = .07).

In the mouse movement condition, the results indicate the acceleration in no-go trials to have a significant relationship with both impulsivity measures – Impulsivity/Emotional lability (*B* = 0.04, *t*(84) = 3.34, *p* <.001), explaining 19 % of variance (*F*(3,84) = 6.43, *p* <.001, *R*^2^ = .19, *R*^2^_adj_ = .16) and DSM-IV: Hyperactive/Impulsive symptoms (*B* = 0.04, *t*(87) = 7.19, *p* = .009), explaining 8% of variance (*F*(1,87) = 7.19, *p* =.009, *R*^2^ = .08, *R*^2^_adj_=.07). Acceleration in no-go trials has also been found to have a significant relationship with ADHD index (*B* = 0.03, *t*(87) = 2.02, *p* =.046). Acceleration in no-go trials explained only 5% of variance in ADHD index scores (*F*(1, 87) = 4.08, *p*=.046, *R*^2^ = .05, *R*^2^_adj_ = .03).

# Supplementary Figures and Tables

## Supplementary Figures

**
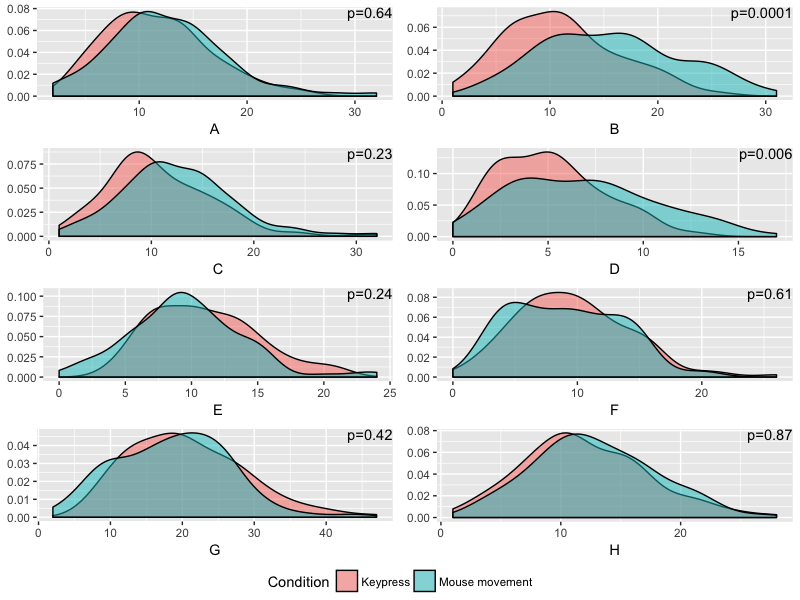
**

**Supplemental Figure 1. Distributions of CAARS scores.** P-values indicate the results of Kolmogorov-Smirnov test. Distributions are plotted using local regression (LOESS) smoothing algorithm. LOESS is a non-parametric method in which simple least squares regression models are fit to the segments of data (Cleveland and Devlin, 1988).


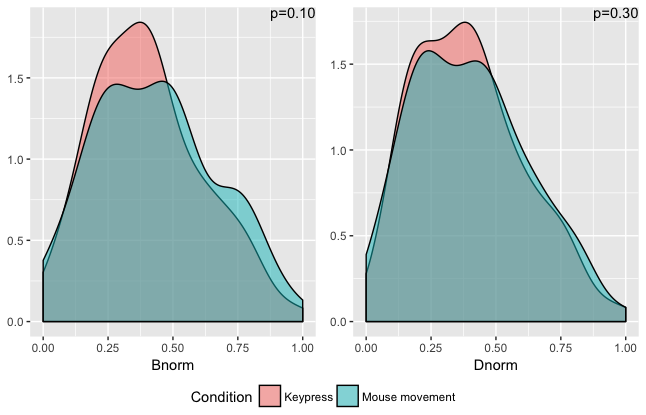


**Supplemental Figure 2. Distributions of min-max-transformed scores**. P-values indicate the results of Kolmogorov-Smirnov test. Distributions are plotted using local regression (LOESS) smoothing algorithm.

## Supplementary Tables

To make sure the distinction between the key press and mouse movement conditions holds for “non-clinical” participants, we reanalyzed Spearman’s rank correlation after removing participants whose CAARS scores exceeded the threshold level specified in Table 2

**Supplemental Table 1**. Spearman’s correlations between log-transformed mouse movement measures and log-transformed CAARS scores with scores above cutoff on subscale C removed (N individuals removed = 3).

|  | Impulsivity/Emotional lability | Problems with self-concept | DSM-IV: Hyperactive-Impulsive Symptoms | DSM-IV: Inattentive symptoms | DSM-IV: ADHD symptoms total |
| --- | --- | --- | --- | --- | --- |
| Velocity in go trials | 0.201 | 0.076 | 0.116 | 0.067 | 0.11 |
| Velocity in No-go trials | 0.355** | 0.109 | 0.272* | 0.197 | 0.228 |
| Acceleration in go trials | 0.194 | 0.08 | 0.103 | 0.06 | 0.101 |
| Acceleration in No-go trials | 0.348** | 0.091 | 0.261* | 0.195 | 0.223 |
| Total distance in go trials | 0.094 | 0.034 | 0.121 | -0.027 | 0.032 |
| Total distance in No-go trials | 0.343** | 0.089 | 0.275* | 0.162 | 0.126 |
| * *p*≤.05, ** *p*≤.01. | | | | | |

**Supplemental Table 2**. Spearman’s correlations between log-transformed mouse movement measures and log-transformed CAARS scores with scores above cutoff on subscale D removed (N individuals removed = 6).

|  | Impulsivity/Emotional lability | Problems with self-concept | DSM-IV: Hyperactive-Impulsive Symptoms | DSM-IV: Inattentive symptoms | DSM-IV: ADHD symptoms total |
| --- | --- | --- | --- | --- | --- |
| Velocity in go trials | 0.166 | 0.089 | 0.053 | 0.048 | 0.05 |
| Velocity in No-go trials | 0.326** | 0.066 | 0.237* | 0.2 | 0.224* |
| Acceleration in go trials | 0.171 | 0.101 | 0.058 | 0.06 | 0.058 |
| Acceleration in No-go trials | 0.322** | 0.05 | 0.233* | 0.203 | 0.224* |
| Total distance in go trials | 0.042 | 0.042 | 0.076 | -0.027 | -0.004 |
| Total distance in No-go trials | 0.304** | 0.055 | 0.217* | 0.157 | 0.191 |
| * *p*≤.05, ** *p*≤.01. | | | | | |

**Supplemental Table 3.** Spearman’s correlations between log-transformed mouse movement measures and log-transformed CAARS scores with scores above cutoff on subscale E removed (N individuals removed = 18).

|  | Impulsivity/Emotional lability | Problems with self-concept | DSM-IV: Hyperactive-Impulsive Symptoms | DSM-IV: Inattentive symptoms | DSM-IV: ADHD symptoms total |
| --- | --- | --- | --- | --- | --- |
| Velocity in go trials | 0.15 | 0.179 | 0.033 | 0.071 | 0.109 |
| Velocity in No-go trials | 0.314** | 0.112 | 0.191 | 0.259; | 0.136 |
| Acceleration in go trials | 0.148 | 0.18 | 0.029 | 0.068 | 0.1 |
| Acceleration in No-go trials | 0.301* | 0.095 | 0.177 | 0.25; | 0.125 |
| Total distance in go trials | 0.074 | 0.118 | 0.07 | 0.106 | 0.18 |
| Total distance in No-go trials | 0.305* | 0.097 | 0.17 | 0.214 | 0.141 |
| * *p*≤.05, ** *p*≤.01. | | | | | |

**Supplemental Table 4.** Spearman’s correlations between log-transformed mouse movement measures and log-transformed CAARS scores with scores above cutoff on subscale F removed (N individuals removed = 8).

|  | Impulsivity/Emotional lability | Problems with self-concept | DSM-IV: Hyperactive-Impulsive Symptoms | DSM-IV: Inattentive symptoms | DSM-IV: ADHD symptoms total |
| --- | --- | --- | --- | --- | --- |
| Velocity in go trials | 0.15 | 0.179 | 0.033 | 0.071 | 0.109 |
| Velocity in No-go trials | 0.314** | 0.112 | 0.191 | 0.259; | 0.136 |
| Acceleration in go trials | 0.148 | 0.18 | 0.029 | 0.068 | 0.1 |
| Acceleration in No-go trials | 0.301* | 0.095 | 0.177 | 0.25; | 0.125 |
| Total distance in go trials | 0.074 | 0.118 | 0.07 | 0.106 | 0.18 |
| Total distance in No-go trials | 0.305* | 0.097 | 0.17 | 0.214 | 0.141 |
| * *p*≤.05, ** *p*≤.01. | | | | | |

**Supplemental Table 5**. Spearman’s correlations between log-transformed mouse movement measures and log-transformed CAARS scores with scores above cutoff on subscale G removed (N individuals removed = 15).

|  | Impulsivity/Emotional lability | Problems with self-concept | DSM-IV: Hyperactive-Impulsive Symptoms | DSM-IV: Inattentive symptoms | DSM-IV: ADHD symptoms total |
| --- | --- | --- | --- | --- | --- |
| Velocity in go trials | 0.201 | 0.076 | 0.071 | 0.067 | 0.11 |
| Velocity in No-go trials | 0.355** | 0.109 | 0.272* | 0.197 | 0.228 |
| Acceleration in go trials | 0.194 | 0.08 | 0.103 | 0.066 | 0.101 |
| Acceleration in No-go trials | 0.348** | 0.091 | 0.261* | 0.195 | 0.223 |
| Total distance in go trials | 0.094 | 0.034 | 0.121 | -0.027 | 0.18 |
| Total distance in No-go trials | 0.343* | 0.089 | 0.275* | 0.162 | 0.141 |
| * *p*≤.05, ** *p*≤.01. | | | | | |
